# The Case for Primary Salivary Rhabdomyosarcoma

**DOI:** 10.3389/fonc.2015.00074

**Published:** 2015-04-01

**Authors:** Mathew Geltzeiler, Guangheng Li, Jinu Abraham, Charles Keller

**Affiliations:** ^1^Department of Otolaryngology – Head and Neck Surgery, Oregon Health & Science University, Portland, OR, USA; ^2^Pediatric Cancer Biology Program, Department of Pediatrics, Papé Family Pediatric Research Institute, Oregon Health & Science University, Portland, OR, USA; ^3^Children’s Cancer Therapy Development Institute, Fort Collins, CO, USA

**Keywords:** sarcoma, rhabdomyosarcoma, head and neck oncology, salivary gland pathology, salivary gland, tumor biology, oncogenesis

## Abstract

Rhabdomyosarcomas of the parotid and submandibular glands have the histological appearance of a skeletal muscle tumor yet can be found in tissue with no striated muscular elements. We examine the potential cell-of-origin for rhabdomyosarcoma and whether salivary tumors represent primary malignancy or metastasis. We have previously established genetically engineered mouse models of rhabdomyosarcoma. In these mice, rhabdomyosarcoma is only induced when a *Pax3:Foxo1* fusion oncogene is activated with concurrent loss of *p53* function (for alveolar rhabdomyosarcoma) or loss of *p53* function alone (for embryonal rhabdomyosarcoma) using Cre-lox technology. These mutations are only activated under the control of promoters specific for selected cell lineages, previously thought to be myogenesis-restricted. RT-PCR and immunohistochemistry for lineage-specific promoter gene products reveal these promoters are active in wild-type mouse salivary gland. Given that mouse rhabdomyosarcoma frequently originates in the salivary glands and these myogenic-related promoters are normally expressed in salivary tissue, a high likelihood exists that the salivary gland contains a cell-of-origin of this muscle-related cancer.

## Introduction

Rhabdomyosarcomas (RMS) of the parotid and submandibular glands are the most lethal forms of salivary gland malignancy in children (1). An analysis of the surveillance, epidemiology, and end results (SEER) database from 1988 to 2001 of parotid or submandibular tumors in patients under 18 years old revealed that 8% tumors were RMS, yet RMS accounted for the majority of salivary malignancy related deaths (1). Salivary gland RMS are of the embryonal subtype (eRMS, 66%), the alveolar subtype (aRMS, 20%), or rarer histologies (2). Seventy percent of aRMS harbor the *t*(2;13) balanced translocation that creates the *Pax3:Foxo1* fusion oncogene (3). In eRMS, no single driving oncogenic mutation has been identified although p53 loss of function is common (4). The salivary glands are also known sites of metastasis for RMS (2). This epidemiology begs the question, is an isolated parotid or submandibular gland RMS a primary salivary gland malignancy, local invasion, or metastasis?

## Materials and Methods

### Animal studies

All studies were performed under institutional Oregon Health & Science University IACUC approval. All mouse lines have been previously described (4, 5).

### Histology and immunohistochemistry studies

The tissue samples were harvested after euthanasia and treated with CRYO-GEL Embedding Medium (Cancer Diagnostics), rapidly frozen in precooled 2-methylbutane (Sigma), and stored at −80°C. For hematoxylin and eosin staining, frozen sections were fixed in 10% formalin for 10 min and were rinsed in distilled water three times then stained with hematoxylin and eosin as previously described (5). For immunostaining, frozen sections were fixed by cold methanol at −20°C and then processed for Pax7 and Myf6 staining as suggested in the manufacturer’s protocol (PK2200, SK 4105, Vector Laboratories and PI-1000, SK 4105, Vector Laboratories, respectively). The anti-Pax7 antibody (Developmental Studies Hybridoma Bank, Iowa City, IA, USA) was used at 1:50 dilution, and the Myf6 antibody (Developmental Studies Hybridoma Bank, Iowa City, IA, USA) was used at 1:200 dilution. Sections were counterstained with hematoxylin. Normal mouse skeletal muscle section was used as the positive control, whereas muscle sections without primary antibody (e.g., secondary only) was set as the negative control.

### RNA isolation and quantitative RT-PCR

Total RNA isolation, cDNA synthesis and RT-PCR for *Pax7*, *Myf5*, and *Myf6* in various mouse organs was performed as described previously (6). The relative expression of *Pax7*, *Myf5*, and *Myf6* was determined by quantitative RT-PCR using Taqman primer and probesets (mouse *Pax7*-Mm00834032_m1, mouse *Myf5*-Mm00435125_m1, and mouse *Myf6*-Mm00435126_m1) on a StepOnePlus™ Realtime PCR system from Applied Biosystems.

## Results

**Figure 1:**
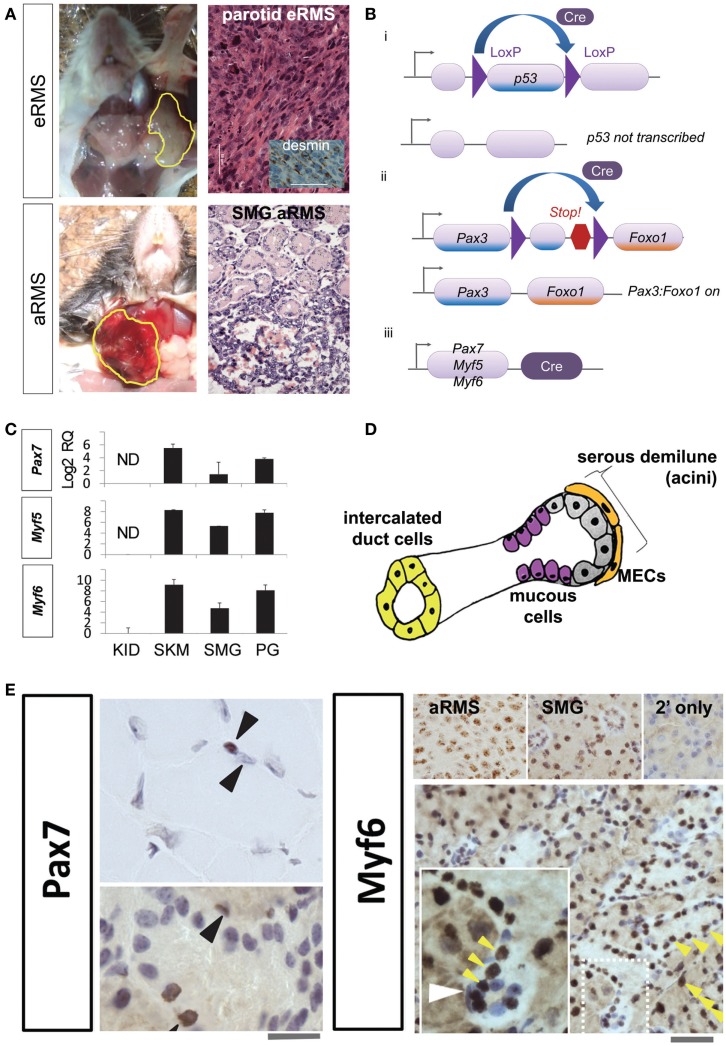
**(A)** Mouse models of salivary gland rhabdomyosarcoma. Top row, representative gross photograph and histologic examination of a mouse parotid embryonal rhabdomyosarcoma: inset, desmin immunohistochemistry. Lower row, representative gross photograph and histologic examination of a mouse submandibular alveolar rhabdomyosarcoma. Scale bar, 50 μm. **(B)** Conditional alleles for rhabdomyosarcoma mouse models. The Cre-LoxP recombination is utilized to activate mutations necessary for RMS tumorigenesis. (i) LoxP sites are inserted on either side of *p53*. Cre recombinase enzyme can then splice out and inactivate (conditionally knock-out) this tumor suppressor gene. (ii) LoxP sites are placed between *Pax3* and an artificially embedded 3′ *Foxo1* gene fragment for this conditional knock-in. Cre can then remove the intervening DNA and generate the *Pax3:Foxo1* fusion oncogene. (iii) Cre recombinase expression is under the control of lineage-specific promoters *Pax7*, *Myf5*, and *Myf6*. **(C)** Expression of myogenic markers in the salivary gland. RT-PCR reveals strong *Pax7*, *Myf5*, and *Myf6* expression in the salivary glands and skeletal muscle satellite cells (but not myotube nuclei), and not in renal tissue. **(D)** Diagrammatic representation of major cell types of the salivary gland. **(E)** Immunohistochemistry of Pax7 in muscle (top left panel) demonstrates strong expression in the skeletal muscle satellite cell nuclei (brown nucleus, upper arrowhead) but not myotube nuclei (non-brown nucleus, lower arrowhead). The submandibular gland also show numerous cells staining positive for Pax7 (bottom left panel; 10 μM scale bar) and Myf6 (right bottom panel; 50 μm scale bar). Myf6+ cells are numerous (yellow arrowheads); however, in the inset a selected slender myoepithelial cells is Myf6 negative (white arrowhead). Abbreviations: eRMS, embryonal rhabdomyosarcoma; aRMS, alveolar rhabdomyosarcoma; SMG, submandibular gland, RMS, rhabdomyosarcoma; RQ, relative quantification; ND, no detectable expression; KID, kidney; SKM, skeletal muscle; PG, parotid gland; MEC, myoepithelial cell.

We have previously created and characterized genetically engineered mouse models (GEMMs) of aRMS and eRMS that are representative of the human diseases and may shed light on the origin of salivary RMS (Figure 1A) (3, 4). In our GEMMs, events activating *Pax3:Foxo1* and/or turning off *p53* are spatially and temporally restricted to a chosen cell lineage by Cre-Lox recombination (Figure 1B). aRMS is only produced if the *Pax3:Foxo1* fusion oncogene is activated with a concurrent loss of p53 function (3). Embryonal RMS requires inactivation of *p53* alone (4). Our mice develop histologically and clinically analogous tumors to the human diseases, and RMS arises from the submandibular or parotid gland in 20% of their tumors (for aRMS and eRMS equivalently).

Historically, we had chosen to express Cre-Lox under promoters active in muscle development and postnatal muscle, namely *Pax7*, *Myf5*, and *Myf6* (Figure 1Biii), based on the observation that RMS is a malignancy with a muscle-related histopathology. A Pax7-Myf5-Myf6 ontogeny is found in a surprising variety of tissues, including not only skeletal muscle but also the pituitary (5–8). In the case of muscle, Pax7+ stem cells (also called satellite cells) remain quiescent until activated, then express Myf5 and divide asymmetrically producing Myf6 expressing myoblast intermediate progenitors, which later become terminally differentiated non-dividing myotubes (5, 8). The related but distinct pituitary ontogeny was first described by our laboratory, whereby Nestin expressing pituitary stem cells give rise to Pax7+ intermediate progenitors, which become Myf6+ terminally differentiated corticotrophs (7). Thus, a Pax7-Myf5-Myf6 ontogeny exists in both muscle and pituitary, but Pax7 is not always expressed in the stem cell (Pax7 can be shifted down in the ontogeny). The most interesting aspect of our studies was that Myf6+ terminally differentiated corticotrophs could have ongoing cell division, which is not seen in terminally differentiated myotubes (7). Given the frequency of salivary RMS in our mouse models, we speculated – and then found the evidence below – that the *Pax7-Myf5-Myf6* ontogeny is also found in native salivary cells, which would logically predispose this tissue to rhabdomyosarcomagenesis.

Development of the mouse submandibular gland begins at E11.5 and continues beyond E15 (9). Embryonically, the majority of submandibular gland mesenchymal cells are derived from the Wnt1-derived cranial neural crest but whether these cranial neural crest cells share expression of Pax3, Pax7, or Myf6 remains yet unexplored (10). In the mature submandibular gland (Figure 1D), well-differentiated myoepithelial cells have ongoing mitotic division (11) – making these cells a potential candidate for transformation. Mesoangioblasts are present in fetal (and to a lesser extent adult) large and small blood vessels, and have the plasticity to become/repair skeletal muscle (12). Interestingly, these mesoangioblasts express Pdgfra (12), which is also a marker of the developing cranial neural crest derived submandibular gland mesenchyme and a key factor in ductal branching morphogenesis (10). Thus, our hypothesis became that the cell-of-origin for rhabdomyosarcoma in the salivary gland is a myoepithelial cell, a mesoangioblast, a retained primitive prenatal mesenchymal cell – or perhaps a salivary cell subtype that is terminally differentiated but comes out of quiescence following transformation. This hypothesis is supported by our findings that many cell types of the adult salivary glands are sometimes Pax7+ and very broadly Myf6+ (Figures 1C,E). For these studies, we performed RT-PCR and immunohistochemistry on mouse submandibular gland tissue of wild-type mice (not harboring any mutation). RNA expression of *Pax7*, *Myf5*, and *Myf6* transcription factors is present in parotid and submandibular gland tissue (Figure 1C). The expression levels of these factors are similar to skeletal muscle. The negative control of the kidney tissue gives reassurance that these transcription factors are not constitutively active in all tissue. By immunohistochemistry (Figure 1E), *Pax7* and *Myf6* are expressed in multiple cell types throughout normal mouse salivary gland tissue including serous demilune cells, mucous cells, and intercalated duct cells – but, surprisingly, not always in myoepithelial cells (Figure 1E white arrowhead). Given that 20% of our mouse RMS tumors originate in the salivary gland and that this tissue inherently utilizes the lineage-specific promoters, which yield aRMS and eRMS, a high likelihood exists that one or more subtype of native salivary cells themselves may represent the cell-of-origin of these tumors.

## Discussion

While our data do not provide definitive evidence that native salivary gland tissue hosts the cell-of-origin for salivary RMS, we do show that the lineages needed to cause mouse RMS are present and actively expressed within salivary tissue. The belief that salivary RMS is the result of metastasis or local invasion might not always be the case. To this end, RMS can also be viewed as a tumor of primitive mesenchyme, which demonstrates a tendency toward myogenesis (13). Cell types of interest for prioritized future studies include myoepithelial cells, mesoangioblasts, and/or retained primitive prenatal mesenchymal cells. Interestingly, primary synovial sarcomas, which can be induced experimentally in mice under a *Myf5* promoter (14), have been reported in the human pancreas (15) – another exocrine gland. Future studies will address the salivary cell subtypes belonging to the *Pax7*, *Myf5*, and *Myf6* lineages, timing of tumor initiation, and the potential stem-cell like plasticity of this ontogeny. A great deal remains to be learned about the cell-of-origin of rhabdomyosarcoma and other cancers; however, given that the cell-of-origin for a tumor determines tumor cell phenotype and possibly treatment paradigms (16), we may soon be empowered by new tools targeting the epigenetic memory of tumors.

## Author Contributions

GH and JA performed experimental studies. CK supervised the studies. MG, GH, and CK wrote the manuscript.

## Conflict of Interest Statement

The authors declare that the research was conducted in the absence of any commercial or financial relationships that could be construed as a potential conflict of interest.
